# Experimental Study on Seismic Performance of Precast Columns Repaired with CFRP Fabrics

**DOI:** 10.3390/ma15217443

**Published:** 2022-10-24

**Authors:** Laijun Liu, Song Lei, Fangwen Wu, Weiwei Lin, Kai Peng, Xiangyan Fan

**Affiliations:** 1 School of Highway, Chang’an University, Xi’an 710064, China; 2 Department of Civil Engineering, Aalto University, 02150 Espoo, Finland

**Keywords:** precast column, rapid repair, CFRP fabrics, cyclic load, seismic performance

## Abstract

Earthquakes worldwide highlight the seismic vulnerability of reinforced concrete (RC) bridge columns. RC bridges are likely to collapse or lose service function due to damage to the bridge columns from strong earthquakes. Rapid repair of RC bridge columns is of great significance for maintaining traffic lines for emergency rescue work after earthquakes. In this study, an improved rapid repair method was developed to restore the bearing capacity of a damaged precast column after earthquake damage. A cyclic loading test was performed to simulate the seismic loading. The original column and the repaired column were both tested. The test results showed that the bearing capacity of the repaired columns was increased by 8%, and the energy dissipation capacity was 53% higher than that of the original column. The ductility decreased because the test for the repaired specimen ended in advance. The initial stiffness of the repaired columns was reduced, but the stiffness was significantly developed in the later loading stage. The rapid repair method proposed in this study exhibited an excellent effect on restoring the seismic resistance of the damaged columns.

## 1. Introduction

There is a considerable portion of aging reinforced concrete bridges that demand rapid and effective methods of repair worldwide. An unnegotiable fact is that the majority of highway bridges were designed according to older design codes. These bridges are vulnerable to disasters such as earthquakes [1]. Frequent earthquakes in recent years (such as the Hualian earthquake (M6.9) in China in 2022, the Michoacán earthquake (M7.5) in Mexico in 2022, and the Luzon Island earthquake (M7.0) in the Philippines in 2022) highlight the role of rehabilitation of damaged bridges to restore their bearing capacity. Rapid repair after a severe earthquake maintains the normal functionality of transportation for rescuing [2], and repair after light earthquakes maintains the service life of aging bridges. As the bridge columns were found to be the critical component of bridges and suffered severe damage from earthquakes [3,4], it is necessary to further develop rapid repair methods for restoring the bearing capacity of reinforced concrete bridge columns.

To restore the bearing capacity of damaged columns, several methods have been developed to provide additional reinforcement, including the enlarging section method [5,6], steel plate method [7,8], steel mesh wrap method [9,10], FRP strip method [11,12], and FRP jacketing method [13,14]. Although steel material and concrete material are easy and cheap to obtain, additional concrete mortar is required in these steel methods. This means it is necessary to wait until the concrete material reaches a certain strength. The cross section of the column is also enlarged, thereby reducing the ductility of the column [15]. Composite polymers have been widely used in building structures recently [16,17,18]. Compared with traditional materials, fiber-reinforced polymers (FRPs) have the advantages of high strength, high ductility, high elastic modulus, and light weight [19,20,21]. Carbon fiber-reinforced polymers (CFRPs) such as CFRP fabrics have been confirmed to be an effective material for reinforcing concrete columns. By wrapping the CFRP fabrics around the surface, not only the bearing capacity of the column is strengthened, but also the ductility of the column is improved [22].

A great number of studies have been conducted on the seismic performance of CFRP fabric-reinforced columns [23,24,25,26,27]. In most of them, the CFRP fabrics were fully warped along the height of the column to make better use of the excellent mechanical performance of the CFRP fabrics. Wang et al. [23] designed a new form of wrapping with gaps and compared its seismic performance with the fully wrapped columns by conducting cyclic loading tests. The results showed that the seismic performance of the fully wrapped columns was increased with respect to the bearing capacity, the ductility, and the energy dissipation capacity. However, Wang et al. [24] found that it was more economical to wrap the column in the plastic hinge region. They designed 11 large-scale square columns and subjected them to cyclic loading tests; the test results showed that the reinforcement effect was more significant when the CFRP fabrics were applied in the potential plastic hinge regions. They also suggested a minimum reinforcing height of 1.1 times the cross-section diameter in later research [25]. Peng et al. [26] compared the repair effects of CFRP fabrics on the different damage levels of columns; the test results indicated that the CFRP fabrics had a better repair effect on severely damaged columns than slightly damaged columns. The effect of the number of CFRP layers was also studied by Elic [27]. Six columns were tested three times with different repair stages, and the results indicated that a single layer of the CFRP fabrics had little effect on the ductility of the repaired column. All studies above have shown that the CFRP fabrics behave well in restoring the seismic performance of bridge columns, including their bearing capacity and ductility. The majority of tested specimens were cast-in-place columns with full-wrapped CFRP fabrics.

Compared with the cast-in-place columns, the precast columns have the advantages of a short construction period, high manufacturing quality, and lower carbon emissions [28]. However, the precast columns are vulnerable to seismic excitation because of the unbonded interface [29]. Precast columns have been widely used in urban bridges and highway bridges in recent years [30], while little research has been conducted on the repair of precast columns after seismic damage. Rapid repair of these bridges after disasters is critical for rehabilitating the functionality of transportation infrastructures. Thus, it is necessary to develop rapid repair methods for precast columns damaged by seismic excitation.

The traditional repair method for the cast-in-place columns does not cover the column–footing interface. However, the column–footing interface is the weakness of the precast column, as discussed above. In this paper, a new repair method with the CFRP fabrics in different directions was specially designed to take into account the weakness of the precast column. The precast column was first tested and damaged under the cyclic loading test. Then, it was repaired with the CFRP fabrics and tested by the cyclic loading test with the same protocol. The experimental results are discussed, including the damage progression, the load–displacement curves, the skeleton curves, the ductility, the stiffness degradation, the energy dissipation, and the measured strain. Finally, the repair effect for the damaged precast column is summarized in the conclusion. This study provides a design for the rapid repair of earthquake-damaged precast columns.

## 2. Experimental Program

### 2.1. Design of Original Precast Column

**Figure 1:**
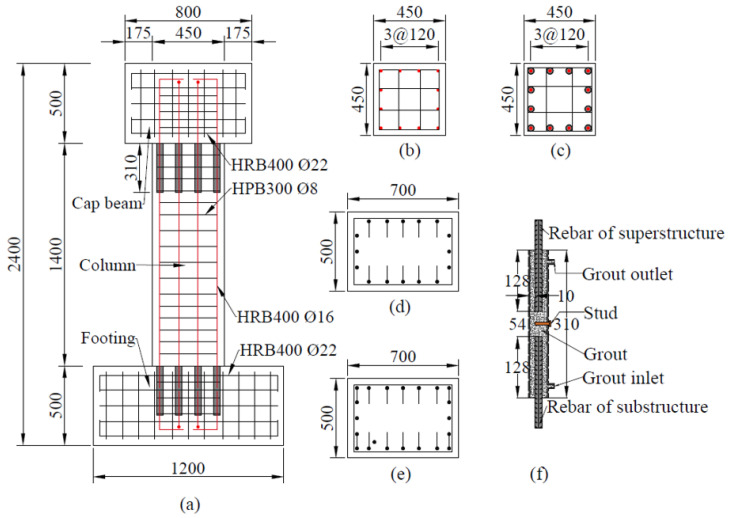
Details of the specimen: (**a**) precast column with grouted sleeve connections; (**b**) cross section of the column; (**c**) cross section of the column with grouted sleeve connections; (**d**) cross section of the cap beam; (**e**) cross section of the footing; (**f**) details of the grouted sleeve.

In order to study the effect of repair with CFRP fabrics on damaged precast columns after earthquakes, a precast column with grouted sleeve connections was designed and tested under cyclic loading. Figure 1 shows the details of the specimen. The specimen used in this test represents a typical precast bridge column. The specimen was divided into three components: the cap beam, the column, and the footing. The column had a height of 1400 mm and a cross section of 450 mm × 450 mm. The aspect ratio of the column was about 3:1. The cap beam had dimensions of 800 mm × 700 mm × 500 mm, and the footing had dimensions of 1200 mm × 700 mm × 500 mm. The reinforcement ratio of the column was 1.19%. The column was longitudinally reinforced by 12-Φ16 HRB400 (hot-rolled ribbed bars with a nominal yield strength of 400 MPa) rebars and transversely reinforced by Φ8 HPB300 (hot-rolled plain bars with a nominal yield strength of 300 MPa) stirrups. The center-to-center spacing of the stirrups was 100 mm in the non-strengthened area and 75 mm in the strengthened area. The cap beam and the footing were reinforced by Φ22 HRB400 rebars, thereby ensuring their failure would occur later than that of the column under vertical and lateral loads. At the cap beam and the footing, longitudinal rebars that extended 150 mm were lap spliced. The grouted sleeve had a height of 310 mm, an inner diameter of 36 mm, and an outer diameter of 42 mm, as shown in Figure 1f. Ultra-high-performance concrete (UHPC) grout material was applied to the grouted sleeve to connect the longitudinal rebars.

Unlike the construction of the cast-in-place column, the components of the precast column were prefabricated individually and then assembled. All components were cured for 28 days before assembly. During the assembling process, cement mortar was poured on the surface of the joint at first. Then, rebars were inserted into the grouted sleeve in different components, and the UHPC grout material was pressed into the grouted sleeves to connect the precast components. The specimen was taken to the cyclic loading test after another 28 days of curing until the grout material gained enough strength.

**Figure 2:**
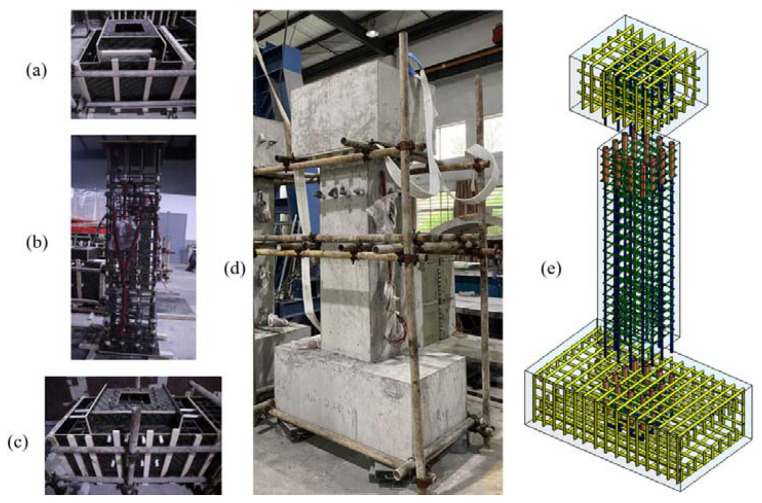
Construction of the precast column with grouted sleeve connections: (**a**) cap beam; (**b**) column; (**c**) footing; (**d**) assembling; (**e**) 3D model of the precast column.

Unlike the construction of the cast-in-place column, the components of the precast column were prefabricated individually and then assembled, as shown in Figure 2. All components were cured for 28 days before assembly. During the assembling process, cement mortar was poured on the surface of the joint at first. Then, rebars were inserted into the grouted sleeve in different components, and the UHPC grout material was pressed into the grouted sleeves to connect the precast components. The specimen was taken to the cyclic loading test after another 28 days of curing until the grout material reached enough strength.

### 2.2. Repair of Damaged Precast Column

Prior to repair, the precast column was tested under vertical load and lateral cycle load to simulate the earthquake excitation. The original precast column was damaged until the lateral load capacity decreased to 85% of the peak load [31]. The test step is described in detail in this paper later, as is the test step for the repaired precast column.

**Figure 3:**
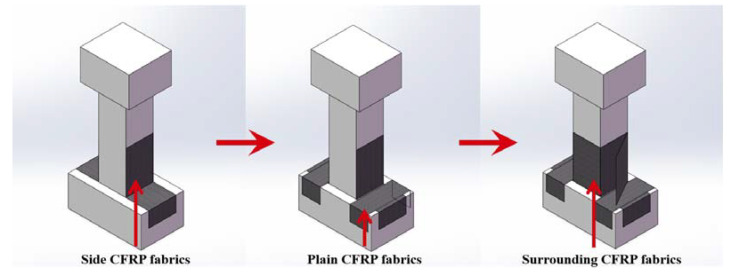
Repair design for the damaged precast column with CFRP fabrics.

After the loading test for the original precast column, the test device was removed, and a repair design was proposed. The design philosophy of the repair for the damaged precast column was to rehabilitate the displacement capacity and strength of the original precast column. Different from the traditional CFRP-confined column design which applies the CFRP fabrics to surround the column [25], side and plain CFRP fabrics were added to the design presented in this paper, as shown in Figure 3. The side CFRP fabrics were applied on the bending surface of the column at first. The flexure behavior of the precast column was enhanced in this way. It is worth noticing that the side CFRP fabrics extend from the column to the footing, thereby strengthening the critical interface of the precast column. Then, the plain CFRP fabrics were applied on the footing to enhance the adhesion of the side CFRP fabrics. Finally, the surrounding CFRP fabrics were applied to the column. The surrounding CFRP fabrics were revealed to improve the mechanical performance of the concrete in the plastic hinge region, including its compressive strength, durability, and ductility [32]. Different fabrics were placed at 0/90 angles in the stack area. The CFRP-confined area was within the height of 700 mm above the column–footing interface, and three layers of the CFRP fabrics were used in each step.

**Figure 4:**
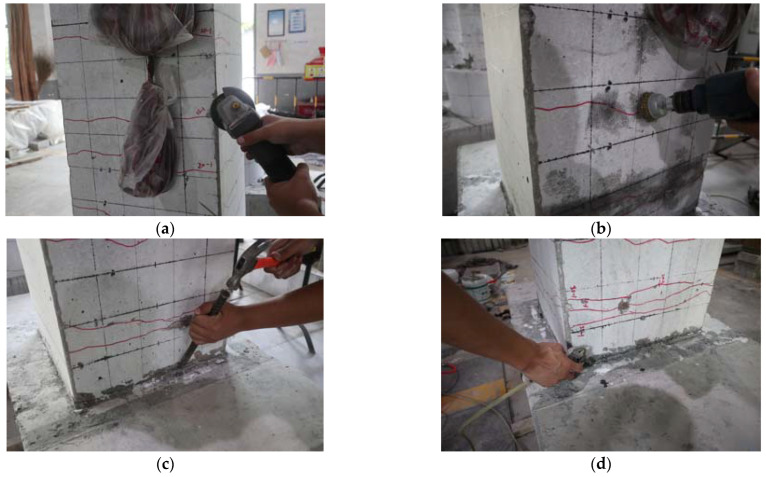

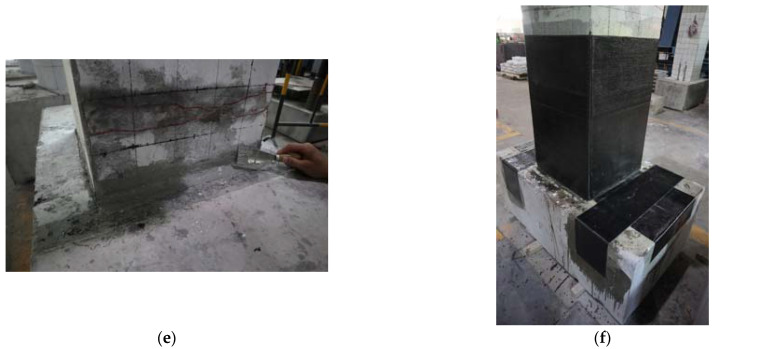
Repair procedures: (**a**) rounding the corner; (**b**) roughing the surface; (**c**) removing the spalling concrete; (**d**) cleaning the surface; (**e**) filling the crack and the corner of the column–footing interface; (**f**) applying the CFRP fabrics.

The repair consisted of the following procedures recommended by CECS 146: 2003 [33], as shown in Figure 4: (a) The sharp corner of the column under the CFRP fabrics was rounded to a radius of 20 mm. The rounded corner prevented the CFRP fabrics from stress concentrations. (b) The surface of the damaged precast column was roughed using an electric metal brush to enhance adhesion to the CFRP fabrics. (c) The spalling concrete in the plastic hinge region was removed using a chisel. (d) The surface of the specimen was cleaned using compressed air. (e) The broken concrete was refilled with epoxy. The corner between the column–footing interface was filled with epoxy to a radius of 20 mm. (f) The CFRP fabrics were applied to the specimen as designed.

### 2.3. Mechanical Properties of Materials

The mechanical properties of all the materials were determined experimentally. The C50 (concrete with a nominal compressive strength of 50 MPa) concrete used in the precast column was tested with three 150 mm cube samples. The tested cubic compressive strength for the C50 concrete was 49.4 MPa after 28 days of curing. The UHPC grout was tested with three 160 mm × 40 mm × 40 mm samples. The tested compressive strength for the UHPC grout was 143 MPa after 28 days of curing.

Three samples of the HRB400 rebars and HPB300 rebars were tested under axial tension. Three samples of grouted sleeve connections were also tested under axial tension. The test results are shown in Table 1.

The thickness of the CFRP fabrics used in this test was 0.167 mm. The test report of mechanical properties was provided by the China National Center Test of Chemical Building Materials. The test results are shown in Table 2.

**Figure 5:**
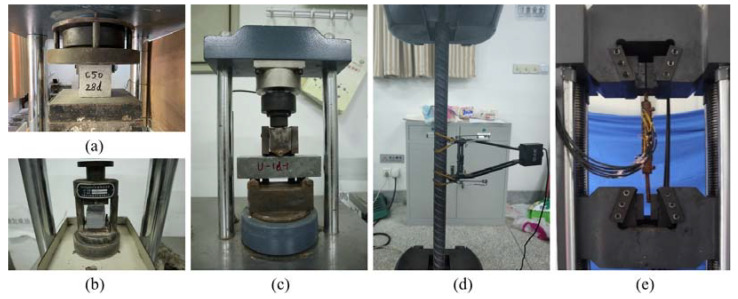
Mechanical property tests: (**a**) concrete; (**b**,**c**) grout; (**d**) rebars; (**e**) grouted sleeve.

All the mechanical properties tests for materials are exhibited in Figure 5.

### 2.4. Instrumentation and Test Step

**Figure 6:**
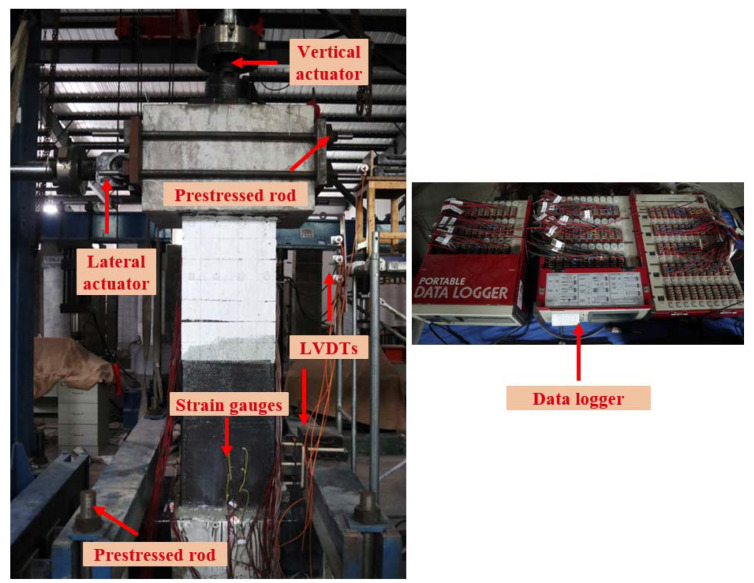
Test step and instrumentation.

**Figure 7:**
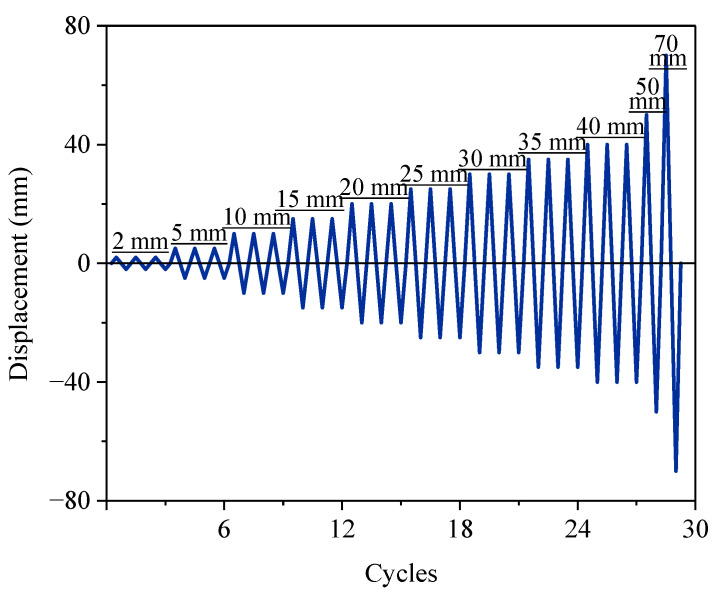
The protocol of the cyclic displacement-control load.

Figure 6 shows the test device for the specimen. A 1000 kN servo-hydraulic vertical actuator was used to apply constant axial pressure on the top of the specimen. The axial pressure was 7% of the specimen’s compressive bearing capacity. A 500 kN servo-hydraulic lateral actuator was used to apply cyclic displacement-control load. The footing was anchored to the ground by the prestressed rod, and the cap beam was anchored with the lateral actuator by the prestressed rod as well. The protocol of the cyclic displacement-control load is shown in Figure 7. Three cycles at each displacement level were conducted until 40 mm. The precast column suffered significant plastic damage at large displacement levels, so one cycle was conducted on the specimen in subsequent displacement levels. During the cyclic loading test on the original precast column, the test ended when the lateral load decreased to 85% of the peak load at a displacement of 66.67 mm. To compare the repaired precast column with the original column, the loading protocol for the repaired specimen was the same as that of the original column, and the test ended after one cycle at the displacement level of 70 mm. The experimental conditions of the tested specimens are shown in Table 3.

Both the vertical actuator and lateral actuator were instrumented with load cells and displacement transformers. The load and displacement data of the actuators were recorded and fed back to control the actuators. Seven linear variable displacement transformers (LVDTs) were instrumented to record the lateral displacements along the height of the specimen. Three LVDTs were spaced 100 mm above the column–footing interface. Another three LVDTs were spaced 100 mm below the cap beam–footing interface. An additional LVDT was installed on the middle height of the cap beam to check the lateral displacement of the actuator. Six strain gauges were used to record the strain of the CFRP fabrics. They were installed on the CFRP fabrics on two perpendicular surfaces. All the measured data were collected by the data logger (TDS-530), as shown in Figure 6.

## 3. Experimental Results and Discussion

### 3.1. Damage Progression

#### 3.1.1. Original Precast Column

**Figure 8:**
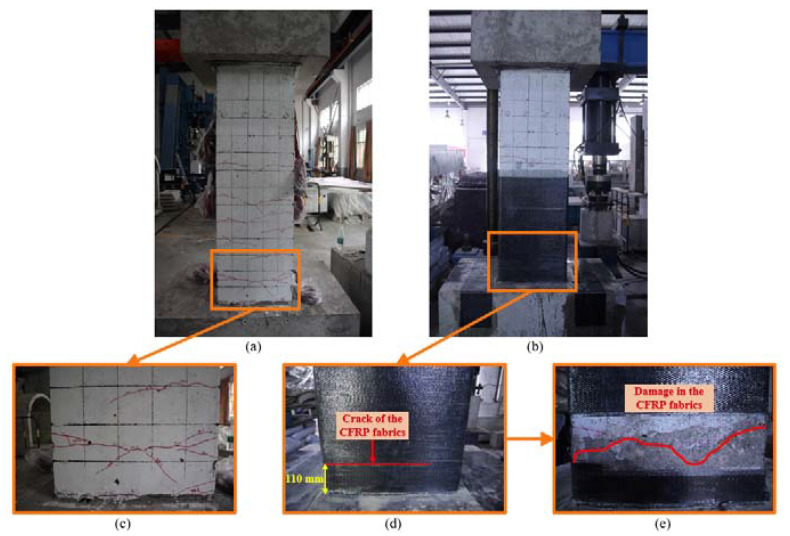
Damage of tested specimens: (**a**) original specimen; (**b**) repaired specimen; (**c**) crack details for the original specimen; (**d**,**e**) crack details for the repaired specimen.

During the cyclic loading test for the original precast column, the specimen exhibited typical flexure–shear damage progression, as shown in Figure 8a. The initial crack appeared about 150 mm and 300 mm above the column–footing interface at the first displacement cycle of 10 mm. Then, evenly distributed flexural cracks appeared within 900 mm above the column–footing interface until 25 mm cycles. The cracks gradually extended and penetrated with the increase in loading cycles. Spalling of concrete was found at displacement cycles of 30 mm and 35 mm. Spalling of concrete concentrated at the corners of the bottom of the column due to the stress concentration here [34]. The cyclic loading test ended when the precast column researched its ultimate capacity at the displacement level of 66.67 mm. The largest crack appeared at the column–footing interface, which was proposed to be a critical interface for the precast column with grouted sleeve connections [35]. Nonetheless, the width of cracks was less than 1 mm during the cyclic loading test for the original precast column.

#### 3.1.2. Repaired Precast Column

**Figure 9:**
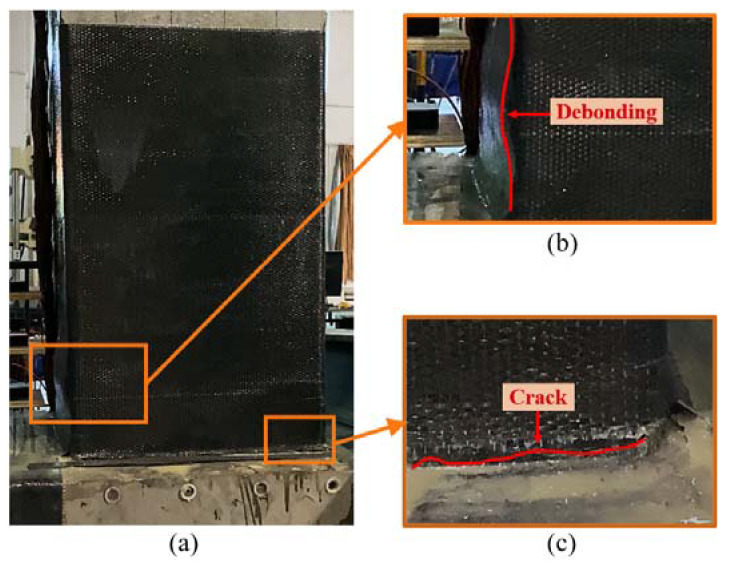
Damage at the displacement level of 70 mm for the repaired specimen: (**a**) damage for the repaired specimen; (**b**) debonding of the CFRP fabrics; (**c**) crack at the column–footing interface.

During the cyclic loading test for the repaired precast column, concrete cracks could not be recorded within the wrap of the CFRP fabrics. Localized debonding was found on the CFRP fabrics within 400 mm above the column–footing interface after the displacement cycles of 15 mm, as shown in Figure 9b. The most significant debonding is 110 mm above the column–footing interface where the CFRP fabrics experienced stress concentration, as shown in Figure 8d. Corresponding to the debonding, a crack formed on the CFRP sheet at the same height, as shown in Figure 8d. It should be noted that a crack could be found at the column–footing interface, as shown in Figure 9c. The width of this crack is larger than that of the crack of the original specimen, but still less than 2 mm. The side fabrics limited the deformation at the column–footing interface.

To compare the damage between the original specimen and the repaired specimen, the CFRP fabrics were peeled off within the debonding area, as shown in Figure 8e. It was found that the protective layer suffered serious damage. The main crack formed at the height of about 150 mm above the column–footing interface. The position of the main crack is the same as that of the initial crack of the original specimen when compared with Figure 8c. Although the column concrete was damaged severely, the bearing capacity was rehabilitated by repair with the CFRP fabrics. The CFRP fabrics also prevented the concrete from spalling.

### 3.2. Load–Displacement Curves

**Figure 10:**
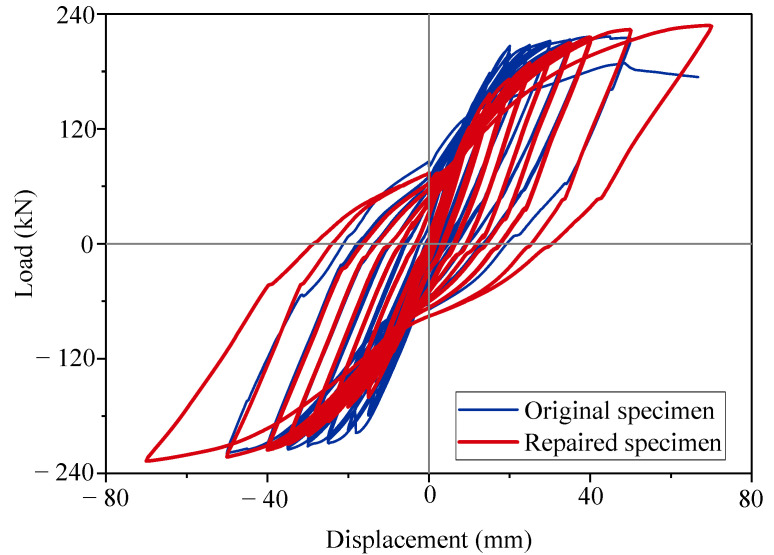
Load–displacement curves.

**Figure 11:**
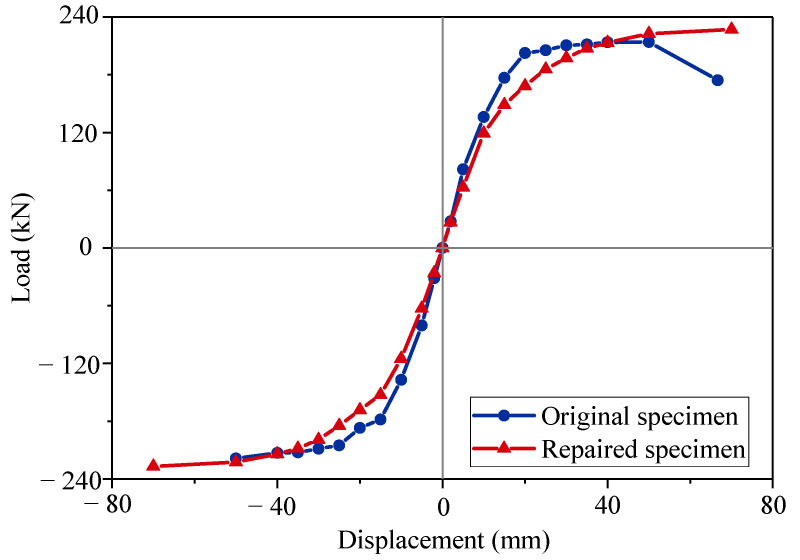
Skeleton curves.

The load–displacement curves reflect the hysteric behavior of the columns under cyclic loading tests. A comparison of the load–displacement curves is shown in Figure 10. The skeleton curves were used to determine the critical characteristics of the seismic performance of the precast columns. The skeleton curves were obtained from the envelope of the load–displacement curves, as shown in Figure 11. Both columns exhibited the pinching effect on the shape of curves as typical reinforcement columns. The original specimen reached the peak point with a peak load (F_p_) of 213.93 kN and a peak displacement (Δ_p_) of 40.1 mm. The repaired specimen had an 8% larger peak load of 227.09 kN at the displacement level of 70 mm. The ultimate strength (F_u_) was 174.19 kN for the original specimen, and the related ultimate displacement (Δ_u_) was 66.67 mm. The ultimate point was the same as the peak point for the repaired specimen. For the repaired specimen, the cyclic loading test ended in advance at the same displacement cycle as the original specimen. The repaired specimen was not at its actual ultimate state, and the actual ultimate displacement should be larger than 70 mm.

Repair with the CFRP fabrics rehabilitated and increased the bearing capacity of the damaged column. However, the initial stiffness of the repaired specimen was obviously lower than that of the original specimen. The repaired specimen also had a larger residual displacement. It was indicated that the concrete column suffered plastic damage before repair. The CFRP fabrics did not work well at lower strains [23]. When the displacement level reached 40 mm, the column experienced enough plastic strain to take advantage of the strong tensile strength of the CFRP fabrics. The repair effect of the CFRP fabrics was limited in the elastic stage, but the repair effort was good in the plastic stage. Some researchers proposed improving the initial stiffness by prestressing the CFRP fabrics [36,37].

**Figure 12:**
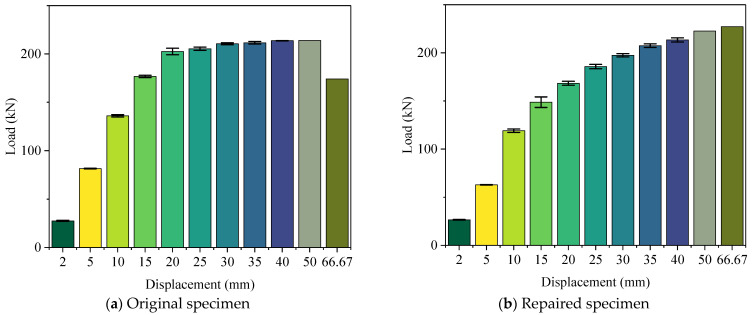
The standard deviation for the peak loads at each displacement level: (**a**) standard deviation of the original specimen; (**b**) standard deviation of the repaired specimen.

It is worth noticing that there were three loading cycles at each displacement level until 40 mm, while there was only one load value at each displacement level shown in Figure 12. The loads in the skeleton curves were the average values of three cycles at each displacement level. In this case, a statistical analysis was conducted, and the standard deviation results are shown in Figure 12.

### 3.3. Ductility

**Figure 13:**
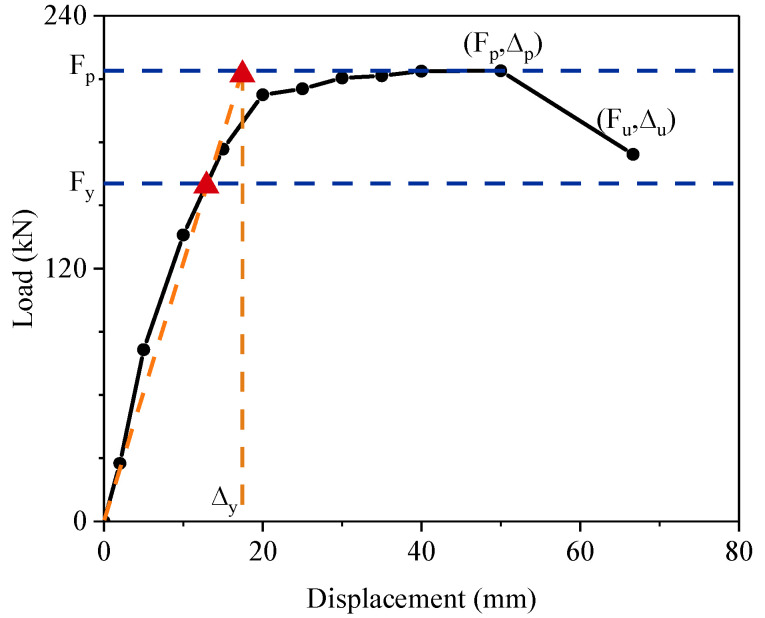
Determination of the yield point according to the Park [38] method.

The yield point was determined by the Park [38] method, which is the method most commonly used to calculate the yield point for reinforcement columns. The principle of the method is shown in Figure 13. The yield load (F_y_) was 0.75 F_p_. Two horizontal lines were drawn at the heights of F_p_ and F_y_. Then, a line was drawn passing the zero point and the intersection point of the skeleton curve and horizontal line F_y_. This line intersected the horizontal line F_p_, and the abscissa of the intersection point is the yield displacement (Δ_y_). The ductility coefficient (μ) is calculated according to Equation (1):

(1) $$\mu =\;\Delta_{\mu }\;/\;\Delta_{y}$$

All the seismic performance characteristics for the specimens are shown in Table 4. The ductility coefficient of the repaired specimen decreased by 11.94% from 3.93 to 3.50. The result was attributed to two reasons: (1) The yield displacement of the repaired specimen had increased by 17.9%. (2) The cyclic loading test of the repaired specimen ended in advance. The repaired precast column had a longer elastic stage than the original specimen. It exhibited larger ultimate displacement and larger loading capacity as well.

### 3.4. Stiffness Degradation

**Figure 14:**
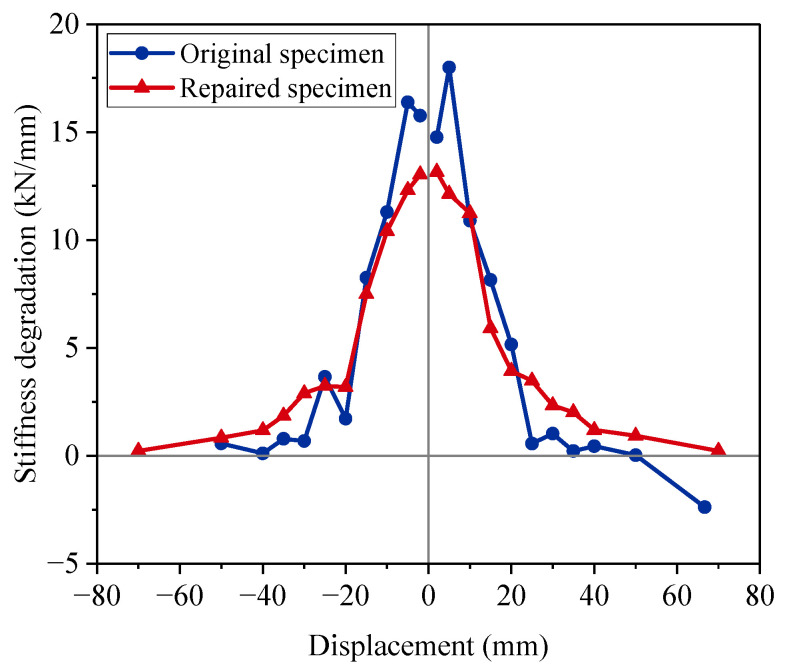
Stiffness degradation.

The stiffness degradation is known as the decrease rate of the bearing capacity [39]. The stiffness degradation for the tested specimens is shown in Figure 14. The stiffness degradation curves were almost symmetrical with minor differences. The construction deviation and material anisotropy contributed to the differences [40]. In the first two displacement levels, the stiffness degradation of the original specimen was much greater than that of the repaired specimen. The maximum error reached 48% at the positive displacement level of 5 mm. For the original specimen, the damage progression for the concrete column developed fast at the primary stage of loading. For the repaired specimen, the concrete was still in a state of low strength with damage. Repair restored the strength of the column by supplementing its strength, rather than helping the original materials regain strength. At the displacement level of 25 mm, the repaired specimen exhibited a decreasing trend in the rate of stiffness degradation, and its stiffness degradation remained larger than the original specimen later. It was indicated that the CFRP fabrics began to exhibit rehabilitation ability. The stiffness degradation of the original specimen was close to 0 at the displacement level of 35 mm. This result showed that the original specimen nearly reached the peak bearing capacity state. The repaired specimen closed to the peak state at the displacement level of 70 mm, which doubled the displacement. The comparison confirmed that the repaired specimen had remaining ductility.

### 3.5. Energy Dissipation

When suffering seismic excitation, structures dissipate seismic energy through flexural displacement. The energy dissipation ability helps structures resist seismic loads, minimizes damage, and prevents structures from collapsing [41].

**Figure 15:**
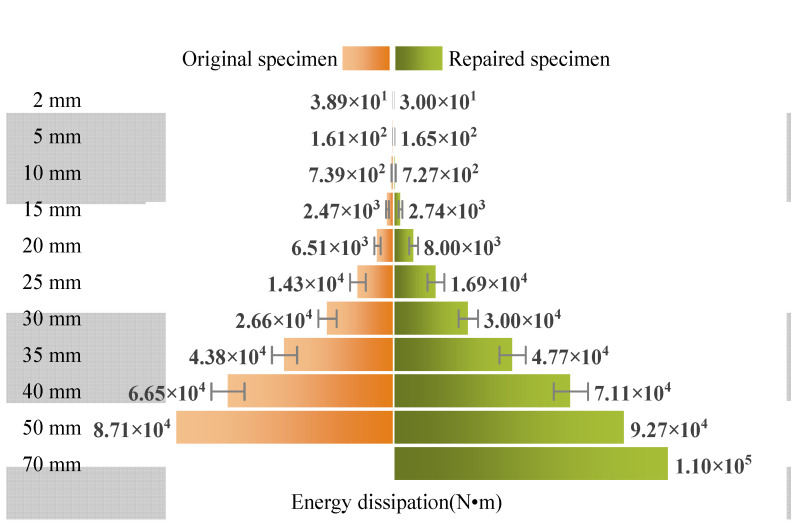
Comparison of energy dissipation at different displacement levels.

Figure 15 shows the energy dissipation at different displacement levels. The data from 2 mm to 40 mm were the average values of three cycles. The standard deviation was also calculated, as shown in Figure 15. The energy dissipation for the original specimen at the displacement level of 66.67 mm could not be calculated because it was not a whole cycle. The repaired specimen showed a similar energy dissipation ability to the original specimen in the first three displacement levels. In the later loading stage, the repaired specimen exhibited better energy dissipation ability. At the displacement cycle of 50 mm, the repaired specimen dissipated 9.27 × 10^4^ N · m of energy. This value was 5.6 × 10^3^ N · m more than that of the original specimen. The energy dissipation ability had not been restored in small displacement levels. However, this ability was significantly strengthened in large displacement levels.

**Figure 16:**
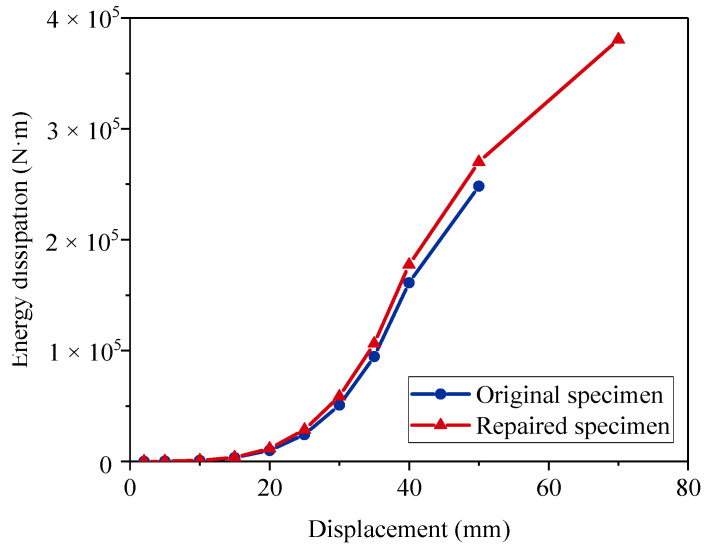
Cumulative energy dissipation.

Figure 16 shows the cumulative energy dissipation. The repaired specimen showed superior energy dissipation ability. The repaired specimen achieved a total energy dissipation of 3.80 × 10^5^ N · m. As the original specimen ended the loading test at the displacement level of 66.67 mm, it was only able to dissipate 2.48 × 10^5^ N · m of energy.

### 3.6. Measured Strain

**Figure 17:**
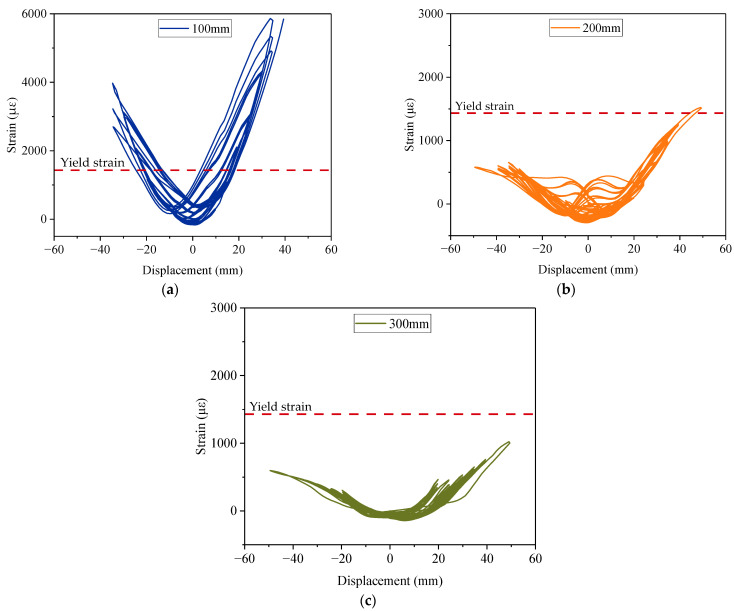
Measured strain of the column surface at different heights: (**a**) 100 mm height; (**b**) 200 mm height; (**c**) 300 mm height.

Strain gauges were instrumented on the surface of the CFRP fabrics to measure the strain of the column at different heights, as shown in Figure 6. The strain of the column surface at the heights of 100 mm, 200 mm, and 300 mm above the column–footing interface was recorded, as shown in Figure 17. The recorded data range was from displacement levels of 2 mm to 50 mm because they were complete cycles. The yield strain was calculated as 1431.88 με.

The surface concrete at the height of 100 mm and 200 mm reached the yield strain at the displacement level of 16.06 mm and 44.06 mm, respectively. The strain at the height of 300 mm did not reach the yield strain during the loading test. The curves of the strain were not strictly symmetrical. In particular, for the one at 100 mm above the column–footing interface, the minimal strain in the load cycles of 35 mm was at the displacement level of −10 mm, as shown in Figure 17a. The column surface experienced obvious residual deformation at this position, which correlated with the debonding and stress concentration of the fabrics as discussed before. The maximum strain at the height of 100 mm reached 5841 με at the displacement level of 39.36 mm in advance. The strain data exceeded the feasible range of the strain gauge later. The maximum strains at the heights of 200 mm and 300 mm were 1522 με and 1024 με, respectively. The particularly high strain at the height of 100 was due to the stress concentration of the CFRP fabrics as well. There was a negative correlation between measured strain stress and the position of the strain gauges.

## 4. Conclusions

A precast column with grouted sleeve connections was experimentally tested in two stages: the original specimen and the repaired specimen. A rapid repair method with the CFRP fabrics was specially designed for the damaged precast column. The test results were discussed to evaluate the effect of repair on the damaged precast column. The following conclusions can be drawn:

1. The repaired specimen suffered severe damage inside the CFRP fabrics but maintained its bearing capacity. The CFRP fabrics prevented the cover concrete from spalling and limited the cracks at the column–footing interface. The main crack of the CFRP fabrics formed at the height of 110 mm above the column–footing interface due to the stress concentration, which was also confirmed by the measured strain.
2. The peak load capacity of the repaired specimen increased by 8%, from 213.93 kN to 227.09 kN. The initial stiffness of the repaired bridge was reduced, but it was significantly developed in the later loading stage. The excellent mechanical performance of the CFRP fabrics was better utilized under high strength state. It was proposed to improve the initial stiffness by prestressing the CFRP fabrics.
3. Although the ductility for the repaired specimen decreased, it was supposed to be larger because the test ended in advance. The stiffness degradation was close to 0 at the displacement level of 70 mm for the repaired specimen, while it was close to 0 at the displacement level of only 35 mm for the original specimen. This confirmed that the repaired specimen had remaining ductility.
4. The energy dissipation capacity was not restored in the early loading stage, but this ability was obviously increased in the later loading stage. The repaired specimen dissipated total energy of 3.80 × 10^5^ N · m, which was 53.31% higher than that of the original specimen.

In future research, prestress load could be applied to CFRP fabrics to strengthen the initial stiffness of the damaged precast columns. Further attempts on prestressed CFRP fabrics could prove quite beneficial for rehabilitating the seismic resistance of damaged precast columns. In addition, the possibility of different fiber-reinforced polymer materials, such as glass fiber-reinforced polymer (GFRP) and basalt fiber-reinforced polymer (BFRP), warrants further investigation.

## Figures and Tables

**Table 1:** Mechanical properties of the rebars and the grouted sleeve.

Type	Yield Strength	Peak Strength
HRB400	481.2 MPa	654.7 MPa
HPB300	360.0 MPa	526.0 MPa
Grouted sleeve	91.6 kN	129 kN

**Table 2:** Mechanical properties of the CFRP fabrics.

Characteristics	Characteristic Values
Tensile strength	3512.7 MPa
Elastic modulus	240,000 MPa
Elongation	1.7%
Bending strength	724.4 MPa
Shear strength	50.4 MPa

**Table 3:** Experimental conditions of tested specimens.

Specimen	Test Arrangement	Axial Pressure Ratio	Repair Method	Thickness of Concrete Protective Layer
Original specimen	1st Tests	7%	-	37 mm
Repaired specimen	2nd Tests	7%	CFRP fabrics	37 mm + 3 layers CFRP

**Table 4:** Seismic performance characteristics for the specimens.

Characteristics	Original Specimen	Repaired Specimen
Yield load	160.45 kN	170.32 kN
Yield displacement	16.96 mm	20.00 mm
Peak load	213.93 kN	227.09 kN
Peak displacement	40.01 mm	70.00 mm
Ultimate load	174.19 kN	227.09 kN
Ultimate displacement	66.67 mm	70.00 mm
Ductility coefficient	3.93	3.50

## Data Availability

The data presented in this study are available on request from the corresponding author. The data are not publicly available due to the funding contract.
